# Early Cognitive Function after Deep Sedation Using Different Anesthetic Agents in Pediatric Patients: A Prospective, Randomized Controlled Trial

**DOI:** 10.3390/medicina60081342

**Published:** 2024-08-18

**Authors:** Min Suk Chae, Ji Yeon Kim, Hyun Jung Koh

**Affiliations:** Department of Anesthesiology and Pain Medicine, Seoul St. Mary’s Hospital, College of Medicine, The Catholic University of Korea, 222, Banpo-daero, Seocho-gu, Seoul 06591, Republic of Korea; shscms@naver.com (M.S.C.); w00dstock@naver.com (J.Y.K.)

**Keywords:** anesthetic agent, cognitive function, inhalation, intravenous, pediatrics, sedation

## Abstract

*Background and Objectives*: The impact of anesthetic agents on memory and cognitive function following general anesthesia is of great interest, particularly regarding their effects on the developing pediatric brain. While numerous studies have examined the relationship between anesthetic drugs and brain function, research focusing on early cognitive function following sedation remains limited. *Materials and Methods*: This study was a prospective, randomized controlled trial involving 148 pediatric patients scheduled for hematological procedures, specifically bone marrow aspiration (BMA) and intrathecal chemotherapy (ITC). Patients were divided into two groups based on the primary anesthetic used: the inhalational sedation group (IHG), in which sevoflurane was used, and the intravenous sedation group (IVG), which received propofol infusion. Apart from the main anesthetic agent, all sedation methods were consistent across both groups. A cognitive function test administered before sedation involved memorizing four distinct images, each associated with a different number. Then, the patients were asked to identify the omitted image upon awakening in the recovery room. Herein, this pre- vs. post-sedation test is called the early recognition assessment (ERA) tool. The primary outcome was the correct response rate after sedation for the two groups. Secondary outcomes included the sedation score, the behavior response score, and the correct response rates according to the number of sedation procedures. *Results*: This study included 130 patients in the final analysis, with 74 originally assigned to each group. The initial cognitive assessment revealed no significant difference in performance between the anesthetic agents. In addition, no differences were observed in the rates of correct responses or post-sedation scores after repeated procedures. However, the IVG demonstrated higher behavior response scores compared to the IHG. *Conclusions*: There were no significant differences in the rates of correct responses using the ERA tool between the two groups, irrespective of the number of sedation procedures performed. While some differences were noted in preoperative, intraoperative, and post-anesthesia care, these did not significantly impact the cognitive outcomes measured.

## 1. Introduction

Concerns persist regarding the potential decline in memory function following general anesthesia, with numerous studies indicating that anesthesia may impact cognitive capabilities across various populations [1,2,3]. These concerns are particularly relevant in pediatric and elderly populations, where cognitive impairments linked to anesthesia have been well documented [3,4,5,6,7]. The increasing need for anesthesia in both age groups, driven by the growing number of surgical and diagnostic procedures, underscores the importance of understanding these potential cognitive effects.

The demand for pediatric anesthesia has risen significantly, primarily due to the necessity of diagnostic and therapeutic procedures that require sedation or general anesthesia. Children with conditions such as hematologic malignancies often require repeated exposure to anesthetic agents over many years for follow-up examinations or treatments, making it crucial to understand the long-term impact of these agents on cognitive and memory functions. Unlike adults, children’s brains are still developing, and the effects of anesthetic agents on brain function during these critical periods of cognitive development could have profound implications. In contrast, elderly patients may experience anesthesia-related cognitive decline, where age-related cognitive function decline and memory loss might confound the effects of anesthesia.

Several studies have suggested that inhaled anesthetics (IHs) might influence brain development, potentially leading to cognitive impairments [8,9,10,11]. Additionally, there is growing concern on cognitive function in children regarding the impact of intravenous anesthetic agents such as midazolam [12,13,14,15], propofol [16,17,18,19], and ketamine [20,21,22].

Cognitive function in pediatric sedation is a concern and has been reported in some cases [23,24]. However, despite these concerns, there is a scarcity of conclusive evidence supporting the impact of various anesthetic agents on cognitive functions during brain development, particularly regarding the immediate cognitive effects following sedation in pediatric patients. The timing of cognitive function assessments post-anesthesia has varied across studies, with differences in assessment time potentially influencing the reported outcomes. This variability has raised questions about the preservation of pre-anesthetic memory after anesthesia and whether early cognitive function of post-anesthesia can be a predictor of long-term cognitive capabilities or memory.

Given these uncertainties, this study aims to investigate whether the choice of anesthetic agents influences early memory function and to identify related factors in children during critical periods of cognitive development. Specifically, this research focuses on pediatric patients with hematologic malignancies, who are frequently exposed to anesthetic agents for sedation due to the necessity of regular follow-up examinations and treatments. This study hypothesizes that the type of anesthetics used may significantly impact early memory function in these patients, which could have implications for their long-term cognitive development.

## 2. Materials and Methods

### 2.1. Ethical Considerations

Approval for this prospective, randomized controlled trial was granted by the Ethics Committee of Seoul St. Mary’s Hospital (KC21OISI0896) on 24 November 2021, adhering to the Declaration of Helsinki principles. This study was registered with the Clinical Research Information Service, Republic of Korea (http://cris.nih.go.kr, KCT0007121) on 25 March 2022. Consent was obtained from all participants through written informed consent prior to enrollment, with participant recruitment occurring between 2 January 2022 and 27 February 2023. The reporting of this study adheres to the CONSORT 2010 Statement and the CONSORT PRO Extension guidelines, with additional details available in the Supplementary Materials.

### 2.2. Study Population

This study included pediatric patients aged 6 to 13 years with hematological malignancies who were undergoing procedural sedation for bone marrow aspiration (BMA) or intrathecal chemotherapy (ITC). Most of them regularly underwent these procedures every three months. Each patient provided informed consent for this study with their guardian before the procedure. Patients who participated in this study must have been literate, have had accurate language skills, and been able to calculate the number. Exclusion criteria included patients with congenital cognitive dysfunction or developmental disabilities, with a history of anaphylaxis or allergies to sedatives, and who were unable to communicate or cooperate due to fear or anxiety before the procedure.

### 2.3. Randomization and Blinding

**Figure 1:**
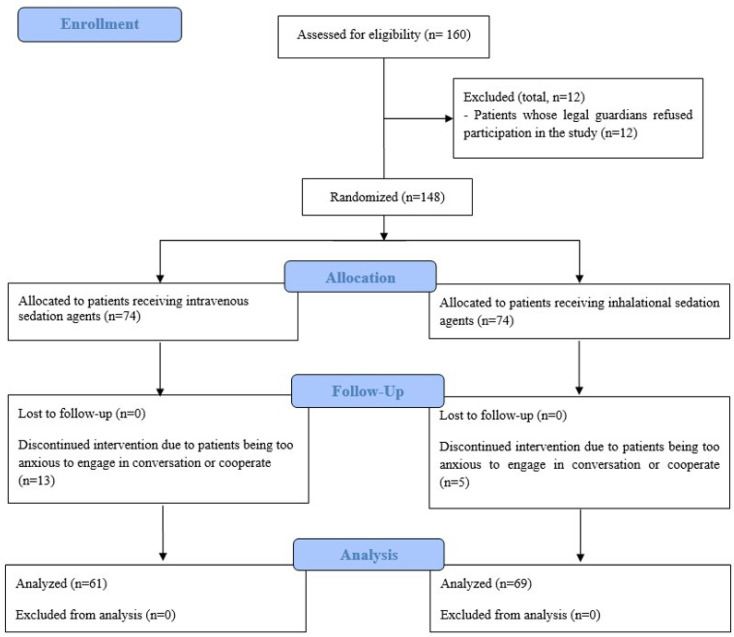
Flow diagram.

Patients were randomly assigned to either the inhalational sedation group (IH-G) or the intravenous sedation group (IV-G) through a stratified block randomization process using a web-based random number generator (www.random.org). A research nurse, uninvolved in the patients’ treatment, oversaw the randomization process. Group assignments were disclosed by opening sequentially numbered, opaque envelopes. Both patients and surgeons were blinded to the group assignments throughout this study. In addition, the medical staff responsible for postoperative care and outcome assessments in the post-anesthesia care unit (PACU) and ward were also blinded to the assignments. The anesthesiologist administering the sedation was informed of the group assignments but did not participate in the postoperative care or outcome evaluations (Figure 1).

### 2.4. Study Protocol

**Figure 2:**
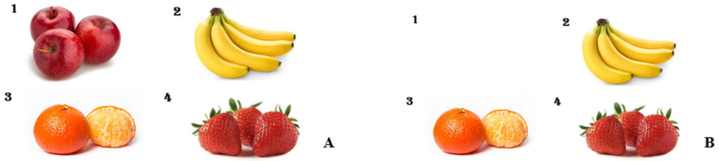
Test pictures. (**A**) Pre-sedation, (**B**) post-sedation.

Prior to the procedure, in the preoperating preparation room, a pre-sedation picture card (Figure 2A) displaying four different fruit images, each with a varying number of fruits, was shown to the patients by medical staff. Each child was asked to name the fruits and count the quantities. They were registered as subjects in this study only after successfully recalling and correctly identifying all images and their corresponding numbers. Following the pre-sedation picture test, patients were transferred to the procedural room. Each patient received 1 mg/kg ketamine for induction and then was maintained on light sedation with mask ventilation. The IHG received 2.5 vol% sevoflurane, while the IVG was administered continuous propofol at a rate of 100 mcg/kg/min. Pain management included administering fentanyl at 1.5 μg/kg for BMA and 1 μg/kg for ITC at the start of the procedures. In cases of movement, the IHG dosage was adjusted to 3.5 vol% sevoflurane with an additional 0.5 μg/kg fentanyl, and for the IVG, propofol was increased to 0.5 mg/kg with 0.5 μg/kg fentanyl. After the procedures, patients were moved to the recovery room and maintained on an oxygen mask until fully awake. In the recovery room, sedation scores (Table 1) and behavioral response scores (Table 2) were evaluated. Upon awakening, a post-sedation test picture (Figure 2B) showing a single omitted fruit was presented to see if patients could recall the missing fruit and its count. A successful recall was marked as a ‘Pass.’ Herein, this pre- vs. post-sedation test is called the early recognition assessment (ERA) tool.

### 2.5. Clinical Variables

Preoperative data collected included the age, type of procedure (BMA or spinal tapping with intrathecal chemotherapy [ST-ITX]), body mass index (BMI), ASA physical class, and number of sedation procedures received. Additional variables included the sedation score and the behavior response score. Intraoperative data included the duration of sedation and the duration of the procedure. Postoperative outcomes measured in the PACU included the sedation score, behavior response score, and time to awaken.

### 2.6. Statistical Analyses and Sample Size

In a preliminary study, the success rates of the ERA tool were 81% for the IVG and 59% for the IHG. To achieve a statistical power of 80%, with a type I error rate < 0.05, and maintaining a 1:1 sample size ratio, our sample size calculation determined that a minimum of 67 patients would be required in each group. Considering an anticipated dropout rate of ~20%, as suggested by preliminary findings, 80 patients per group were enrolled to ensure the reliability and robustness of our trial results.

Continuous variables were assessed for normality using the Shapiro–Wilk test. Data are expressed as means ± standard deviations for normally distributed variables, and as medians with interquartile ranges for non-normally distributed variables. For the analysis, Student’s *t*-tests were used for normally distributed data, while Mann–Whitney U tests were applied to non-normally distributed data. Categorical variables are presented as frequencies and percentages, and comparisons were made using Pearson’s chi-square test or Fisher’s exact test as appropriate. A two-sided *p* value < 0.05 was considered statistically significant. All statistical analyses were conducted using SPSS for Windows (version 24.0; SPSS Inc., Chicago, IL, USA), and graphical representations were created with Microsoft Excel.

## 3. Results

### 3.1. Flow Diagram

Initially, 160 patients were assessed to be eligible for participation. Prior to randomization, 12 were excluded due to their legal guardians’ refusal to participate. Consequently, 148 patients were enrolled and randomized into two groups (IHG: *n* = 74, IVG: *n* = 74). During follow-up, due to anxiety-related non-cooperation, the IHG experienced 5 dropouts and the IVG had 13. Ultimately, 61 patients in the IVG and 69 in the IHG were analyzed (Figure 1).

### 3.2. Comparisons in Preoperative, Intraoperative, and PACU Variables between IVG and IHG (Table 3)

A comparison of preoperative, intraoperative, and PACU variables between IV and IH sedation agents for pediatric procedures is summarized in Table 1. Demographic characteristics, including sex distribution (male and female participants were included in this study, with females comprising 37.7% in the IVG vs. 46.4% in the IHG, *p* = 0.318), were similar between the two groups. No statistically significant difference in sex distribution was found between the two groups. The median age was also comparable, 8.0 years in the IVG and 10.0 years in the IVG (*p* = 0.342), between the two groups. The distribution of procedure types was also comparable, with a bone marrow transplant (57.4% IV vs. 60.9% IH, *p* = 0.686) and intrathecal therapy (42.6% IV vs. 39.1% IH) being the most common. There were no significant differences in etiology between the groups (*p* = 0.974).

Intraoperative variables revealed significant differences in the number of sedation procedures received (*p* < 0.001), with a higher proportion of first-time procedures in the IHG (94.2%) compared to the IVG (63.9%). The durations of sedation and procedures were similar between the groups (*p* = 0.811 and *p* = 0.884, respectively). Sedation maintenance methods varied, with the IVG primarily receiving propofol, ketamine, midazolam, and dexmedetomidine, while the IHG received sevoflurane. There were no significant differences in analgesic opioid consumption between the groups (*p* = 0.187).

PACU variables showed that the median cognitive assessment time was longer in the IHG (9.0 min) than in the IVG (5.0 min, *p* = 0.013). In addition, there were significant differences in the sedation and behavior scores at evaluation (*p* = 0.049 and *p* < 0.001, respectively), with higher sedation and lower behavior scores observed in the IHG.

**Table 3:** Comparisons of preoperative, intraoperative, and PACU variables between IV and IH sedation agents for pediatric procedures.

Group	Intravenous Sedation	Inhalational Sedation	*p* Value
n	61	69	
***Preoperative variables***			
Sex (female)	23 (37.7%)	32 (46.4%)	0.318
Age (years)	8.0 (6.5–11.0)	10.0 (6.5–13.0)	0.342
Procedure types			
Bone marrow transplant	35 (57.4%)	42 (60.9%)	0.686
Intrathecal therapy	26 (42.6%)	27 (39.1%)	
Etiology			
Acute lymphoblastic leukemia	40 (65.6%)	44 (63.8%)	0.974
Acute myeloid leukemia	12 (19.7%)	14 (20.3%)	
Others	9 (14.8%)	11 (15.9%)	
Height (cm)	136.9 (121.8–150.9)	143.5 (121.1–157.1)	0.412
Weight (kg)	36.5 (25.0–50.6)	40.2 (24.0–52.8)	0.755
Body mass index (kg/m^2^)	18.6 (15.9–22.0)	18.8 (16.4–20.9)	0.928
***Intraoperative variables***			
Number of sedation procedures received			
First time	39 (63.9%)	65 (94.2%)	<0.001
Second time	11 (18.0%)	4 (5.8%)	
Third time	11 (18.0%)	0 (0.0%)	
Sedation duration (min)	15.0 (12.0–20.0)	15.0 (11.5–19.5)	0.811
Procedure duration (min)	7.0 (5.0–13.5)	8.0 (5.0–12.0)	0.884
Sedation maintenance during procedures			
Intravenous sedative dosage (mg)			
Propofol	90.0 (60.0–120.0)	N/A	
Inhalational sedative dosage (MAC)			
Sevoflurane	N/A	2.0 (2.0–3.0)	
Analgesic opioid consumption (mcg)			
Fentanyl	30.0 (16.9–50.0)	20.0 (7.5–50.0)	0.187
***Post-anesthesia care unit variables***			
* Cognitive assessment time (min)	5.0 (2.0–15.0)	9.0 (5.0–17.0)	0.013
Sedation score at evaluation (points)	6.0 (6.0–6.0)	6.0 (5.0–6.0)	0.049
Behavior score at evaluation (points)	4.0 (3.0–4.0)	1.0 (1.0–1.0)	<0.001

**Abbreviations:** N/A, non-applicable; MAC, minimum alveolar concentration. The cognitive assessment time is defined as the duration from the arrival in the post-anesthesia care unit to the awakening. Values are expressed as the median (interquartile range) and numbers (proportion). * time to awaken after entering PACU

### 3.3. Comparisons of Correct Response Rates for the ERA Tool between IVG and IHG

**Figure 3:**
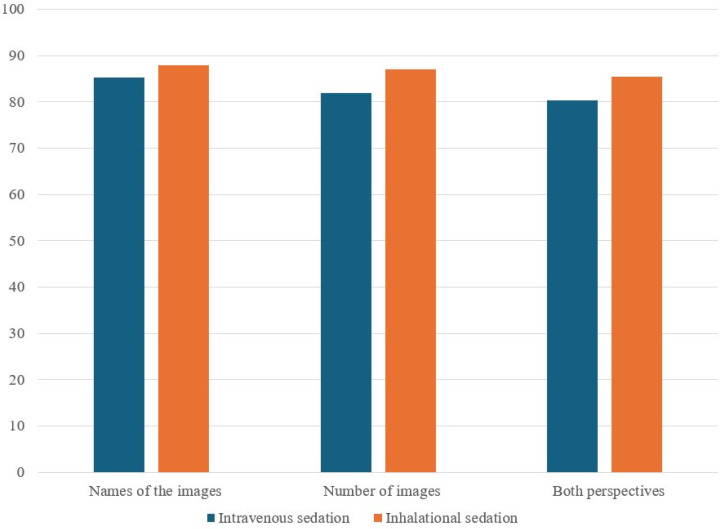
Correct response rates (%) for ERA tool between IVG and IHG in pediatric procedures. Both perspectives are defined as all correct responses of the name and number of images.

Table 4 and Figure 3 detail the correct response rates for the ERA tool between the two groups. The rates for accurately naming images were marginally higher in the IHG (88.4%) compared to the IVG (85.2%) but this difference was not statistically significant (*p* = 0.594). Similarly, the correct response rates for the number of images (87.0% IHG vs. 82.0% IVG, *p* = 0.431) and for both aspects combined (85.5% IHG vs. 80.3% IVG, *p* = 0.432) showed no significant differences.

### 3.4. Comparisons in the Effect of the Number of Sedation Procedures on Correct Response Rates between IVG and IHG

**Figure 4:**
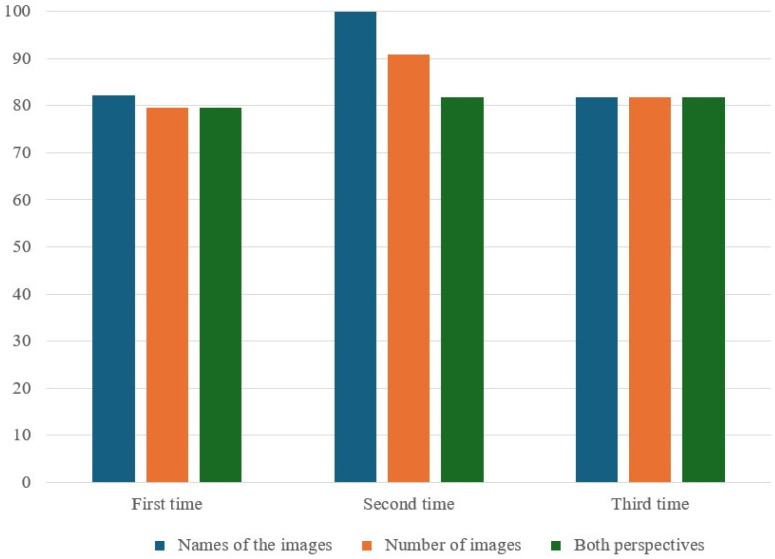
Correct response rates (%) for ERA tool by the number of IV sedation procedures received. Both perspectives are defined as all correct responses of the name and number of images.

The impact of the number of sedation procedures on correct response rates is detailed in Table 5 and Table 6. For the IVG (Table 5 and Figure 4), correct response rates for naming images initially increased from 82.1% in the first procedure to 100.0% in the second procedure, then slightly decreased to 81.8% in the third. This trend was not statistically significant (*p* = 0.313). Similarly, the rates for the number of images and both perspectives showed no significant changes across the different procedural exposures (*p* = 0.685 and *p* = 0.976, respectively).

**Figure 5:**
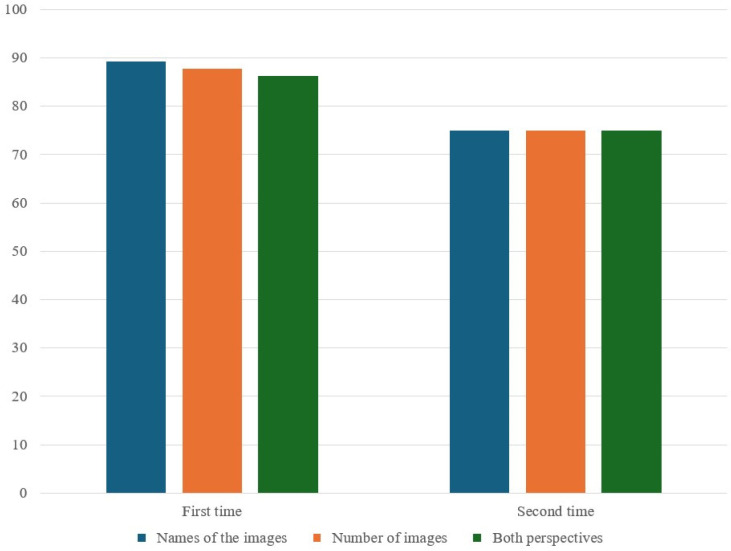
Correct response rates (%) for ERA tool by the number of IH sedation procedures received. Both perspectives are defined as all correct responses of the name and number of images.

In the IHG (Table 6 and Figure 5), the rates for naming images were higher for first-time procedures (89.2%) than for second-time procedures (75.0%), although this difference was not statistically significant (*p* = 0.396). The same trend was observed for counting the number of images (87.7% first time vs. 75.0% second time, *p* = 0.436) and for both aspects combined (86.2% first time vs. 75.0% second time, *p* = 0.474).

## 4. Discussions

This study did not find significant differences in the correct response rates for the ERA tool between IVG and IHG, irrespective of the number of sedation procedures received. While there were some differences in preoperative, intraoperative, and PACU variables (i.e., number of sedation procedures, behavior response scores, and cognitive assessment times post-anesthesia), these did not translate into significant differences in the cognitive outcomes measured using the assessment tool.

The impact of IH anesthetic agents such as sevoflurane on cognitive function has been demonstrated in animal studies. For instance, neonatal exposure to sevoflurane has been shown to affect learning and memory by changing hippocampal DNA methylation [25]. Given that sevoflurane can induce neurotoxicity in various ways within the developing brain, the careful consideration of its usage period and dosage is recommended [11]. In addition, this compound can impair learning and memory by reducing neuronal glucose transporter activity, thus affecting glucose metabolism [10].

However, these effects on neuronal activity have predominantly been studied in nonhuman animals, and applying these findings to humans, who have different developmental trajectories, is challenging. In our study, sevoflurane did not adversely affect early memory function in children, indicating a departure from the patterns observed in animal studies concerning the impact of inhaled anesthetics on the developing brain.

Propofol, a widely used anesthetic for procedural sedation in pediatric patients, has also been scrutinized for its potential effects on brain cells, depending on the dosage and duration of exposure [16,26,27,28,29]. Some studies suggest that while a single, short exposure to propofol has minimal impact on cognitive function, prolonged or recurrent exposures could impair memory or cognitive function, potentially leading to long-term cognitive deficits in humans and other animals [30]. High doses may mitigate the development of postoperative cognitive dysfunction [31]. However, our findings indicate that neither single nor recurrent exposures to propofol resulted in early memory impairment. Although long-term effects were not assessed, our results suggest minimal short-term impact on cognitive abilities from propofol exposure.

Many studies have highlighted the importance of the timing of exposure. The pediatric patients involved in this study were not at the stage of primary brain structure formation, but rather in a phase during which growth of white and gray matter, which governs physical functions and basic behavior patterns, is prevalent. As indicated by our results, short-term exposure to anesthetics in this age group did not significantly affect cognitive function.

Neither IH nor IV anesthetic agents impacted early cognitive function. In addition, despite concerns that inhaled anesthetics might frequently cause emergence hyperactivity [32,33,34,35], we found that the use of IV anesthetics resulted in a higher degree of alertness and greater behavioral response upon awakening. However, these factors did not affect early cognitive function. The differences in behavioral response at awakening seem to be related to the longer awakening times associated with IH anesthetics, although the precise reasons for this remain unclear. Nevertheless, both groups demonstrated a high level of alertness during the ERA test.

There were no differences in early memory impairment with different anesthetics during recurrent exposure. Future studies should explore whether cognitive function changes over time with increased frequency of anesthetic exposure.

This study had some limitations. First, as not all patients underwent the same procedure, variation in the procedure type (BMA or ITC) could influence sedation duration and awakening times. Although the amount and duration of the procedures did not significantly impact the study outcomes, ensuring a uniform patient group undergoing the same procedures would be ideal for future research. Second, the number of subjects undergoing repeat procedures varied between the two groups, limiting the accuracy of the analysis due to differences in exposure frequency. Future studies should analyze equal numbers of exposures and subjects across groups. Third, although we evaluated early cognitive function, assessments were only conducted immediately after the first awakening. To provide a comprehensive evaluation, future research should assess cognitive function at various time points through regular intervals in the early postoperative period.

## 5. Conclusions

There were no significant differences in response results for the ERA tool between the IVG and IHG, irrespective of the number of sedation procedures received. Since no differences in early memory function were observed depending on anesthetic agents, pediatric patients may be free to choose anesthetic agents compared to before; further research involving a larger cohort is needed to thoroughly investigate the effects of anesthetics on cognitive function.

## Figures and Tables

**Table 1:** Sedation score in PACU.

Sedation Score
1. no response to light tapping or shaking
2. slight response to light tapping or shaking
3. only response when their name is called loudly or repeatedly
4. response by blinking eyes when one’s name is called in normal tone
5. response and goes back to sleep when one’s name is called in normal tone
6. response immediately when one’s name is called in anormal tone. They are alert and can make clear decisions

**Abbreviations:** PACU, post-anesthetic care unit.

**Table 2:** Behavior response score in PACU.

Behavior Response Score
1. Stay calm, cooperate, and follow instructions
2. Anxious, but if you advise and comfort them, they will remain calm
3. Anxious, and even when calmed down, they are on the verge of crying, looking for one’s mother
4. Crying, blowing, making a fuss, not staying still, and constantly moving with resistance

**Table 4:** Correct response rates for ERA tool between IVG and IHG in pediatric procedures.

Group	Intravenous Sedation	Inhalational Sedation	*p* Value
n	61	69	
The correct response rate for the early recognition assessment tool			
The names of the images	52 (85.2%)	61 (88.4%)	0.594
The number of images	50 (82.0%)	60 (87.0%)	0.431
* Both perspectives	49 (80.3%)	59 (85.5%)	0.432

* Both perspectives are defined as all correct responses of the name and the number of images. Values are expressed as numbers (proportion).

**Table 5:** Correct response rates for ERA tool by number of IV sedation procedures received.

Number of IV Sedation Procedures Received	First Time	Second Time	Third Time	*p* Value
	n = 39	n = 11	n = 11	
Correct response rate for the ERA tool				
Names of images	32 (82.1%)	11 (100.0%)	9 (81.8%)	0.313
Number of images	31 (79.5%)	10 (90.9%)	9 (81.8%)	0.685
* Both perspectives	31 (79.5%)	9 (81.8%)	9 (81.8%)	0.976

* Both perspectives are defined as having correct responses for both the name and number of images. Values are expressed as numbers (proportion).

**Table 6:** Correct response rates for ERA tool by number of IH sedation procedures received.

Number of IH Sedation Procedures Received	First Time	Second Time	*p* Value
	65	4	
Correct response rate for the ERA tool			
Names of images	58 (89.2%)	3 (75.0%)	0.396
Number of images	57 (87.7%)	3 (75.0%)	0.436
* Both perspectives	56 (86.2%)	3 (75.0%)	0.474

* Both perspectives are defined as having correct responses for both the name and number of images. Values are expressed as numbers (proportion).

## Data Availability

The data supporting the findings of this study are contained within the article.

## Supplementary Materials

The following supporting information can be downloaded at: https://www.mdpi.com/article/10.3390/medicina60081342/s1, Supplementary Material: Textcheck Certificate.
