# Supplementary material for: Early Cognitive Function after Deep Sedation Using Different Anesthetic Agents in Pediatric Patients: A Prospective, Randomized Controlled Trial
Source: Medicina (Kaunas). 2024 Aug 18;60(8):1342. doi: 10.3390/medicina60081342 (PMC11356384; doi:10.3390/medicina60081342)
Supplement: Supplementary file 1 [file medicina-60-01342-s001.zip › medicina-3146920-supplementary.pdf]

Textcheck Certificate

Refnum:	24072410
Title:	Early recognition assessment according to anesthetic agent after deep sedation in pediatric patients; A prospective, randomized controlled trial
Date:	2024/07/26

We hereby certify that Textcheck has checked and corrected the English in the manuscript named above.

A specialist editor with suitable professional knowledge (M.Sc. or Ph.D./M.D.) reviewed and corrected the English. An English language specialist subsequently checked the paper again. The first language of both editors is English.

Please direct any questions regarding this certificate or the English in the certified paper to: certified@textcheck.com (Please quote our reference number: '24072410')
